# Stem cell therapy in Alzheimer’s disease: current status and perspectives

**DOI:** 10.3389/fnins.2024.1440334

**Published:** 2024-11-21

**Authors:** Chu-Min Ou, Wei-Wei Xue, Dong Liu, Liya Ma, Hai-Tao Xie, Ke Ning

**Affiliations:** ^1^Guangdong Celconta Biotechnology Co., Ltd., Dongguan, Guangdong, China; ^2^School of Biological Engineering, Dalian Polytechnic University, Dalian, China; ^3^Sheffield Institute of Translational Neuroscience (SITraN), University of Sheffield, Sheffield, United Kingdom

**Keywords:** Alzheimer’s disease, stem cell therapy, Aβ deposits, nFTS, clinical trials

## Abstract

An *incurable* neurogenerative illness, Alzheimer’s disease, is the cause of most global health, medical, and social disasters. The two main symptoms are cognitive impairment and neuronal loss. Current medications that target tau protein tangles and Aβ plaques are not very effective because they only slow the symptoms of AD and do not repair damaged cells. Stem cell-based treatments, however, present an alternative strategy in the treatment of AD. They have the capacity to divide into specialized adult cells, have self-renewal abilities, and multiplication. Stem cells can now be employed as a donor source for cell therapy due to developments in stem cell technology. This review covers preclinical and clinical updates on studies based on targeting the tau protein tangles and Aβ plaque, as well as four types of stem cells employed in AD treatment. The review also outlines the two basic pathologic aspects, tau protein tangles and Aβ plaques, of AD.

## 1 Introduction

In 2021, WHO posted the seventh most common cause of death globally is Alzheimer’s disease (after Ischaemic heart disease, COVID-19, Stroke, Chronic obstructive pulmonary disease, Lower respiratory infections, Trachea, bronchus, lung cancer), which is a neurological illness linked to advanced progressive dementia. Symptoms of the condition include mood swings, sleep disturbances, behavioral changes, and cognitive impairment. Severe symptoms include malnutrition, neuronal necrosis that results in multiple organ failure, and even brain death as AD progresses to advanced stages (Ma et al., 2022).

Age 65 can be used to distinguish between two types of AD, early-onset EOAD (before age 65) and late-onset LOAD (after age 65), both of which share similar clinical characteristics (Penke et al., 2023). According to estimates, EOAD has a heritability of 90–100% (Wingo et al., 2012), whereas LOAD heritability, which ranges from 60% to 80%, is marginally lower than that of other diseases (Gatz et al., 1997). An important contributing factor to autosomal dominant early-onset Alzheimer’s disease (EOAD) is a mutation in the presenilin-1 (PS-1) gene (Hanisch and Kölmel, 2004). Several genetic loci or polygenic effects work together to produce the genetic composition of LOAD, which makes this disease far more complex than EOAD. According to genetic research, there are multiple components of Alzheimer’s disease, and the most significant risk factor for LOAD (cholesterol synthesis and transport) is ApoE4 (Sims et al., 2020). And we can see the different between FAD and SAD in Table 1.

**TABLE 1:** Sporadic and familial forms of AD.

	Familial AD (FAD)	Sporadic AD (SAD)
Etiology	Genetic factors, and mutations in presenilin-1, presenilin-2, and amyloid precursor protein genes.	Environmental, APOE, aging, and vascular disease, TBI and risk factors associated with diet, the immune system, mitochondrial function, metal exposure, and infection.
Pathogenesis	The mutations within these three genes (PS1/PS2/APP) affect a common pathogenic pathway in APP synthesis and proteolysis, which lead to excessive production of amyloid β.	1) by promoting the liberation of oxygen free radicals with age, 2) via environmental stress acting on regulatory genes early and later in life (‘dual hit’ hypothesis), or 3) by increasing the cumulative ‘allostatic load’ on the body over a lifetime.
Pathophysiology	Increased toxic Aß_42_ initiates a cascade of down-stream pathological processes such as tau hyperphosphorylation, neurofibrillary tangles (NFTs) formation, neuroinflammation, loss of synaptic junctions and neuronal cell death, with dementia being the ultimate clinical result of these progressive neurodegenerative processes.	
Features	Distinctive features including early age at onset, positive familial history, a variety of non-cognitive neurological symptoms and signs, and a more aggressive course.	There is a lack of full understanding of the underlying causes and molecular mechanisms leading to this progressive form of dementia.
References	Bird, 1994; Tehrani et al., 2024; Wu et al., 2012	Armstrong, 2019; Frisoni et al., 2022; Polsinelli and Apostolova, 2022

Owing to the disease’s prevalence, the rising use of memory consults, and advancements in treatment, it is becoming increasingly obvious that Alzheimer’s disease (AD) must be diagnosed early. In the early stages of AD, a variety of strategies have been developed to identify patients who are at risk. It has been suggested that biological markers have been detected in CSF or blood. Considerable research has been done on other anatomical markers, such as the use of nuclear magnetic resonance imaging (MRI) to detect hippocampal atrophy. However, the clinical approach continues to be the most commonly employed in the early diagnosis field. However, it has only been able to identify the earliest cognitive disorders and explain the various phases of memory system alteration associated with the progression of lesions within the hippocampus. Non-cognitive or behavioral deficits are still considered late and are associated with the spread of the lesion to the neocortex, especially to the frontal lobes (Andreasen et al., 2001; Bature et al., 2017; Michel et al., 2010). Involvement of the cerebral cortex involved in higher levels of processing occurs at later stages and eventually much of the brain becomes affected leading to profound consequences in terms of the ability of the individual to lead an independent life.

A novel therapeutic option for AD has been developed based on the remarkable outcomes of stem cell therapies, which have recently been shown in preclinical and clinical trials. In this review, we address treatment approaches for AD-related diseases and discuss new developments in stem cell therapy for AD.

## 2 Characterization of AD pathology

Based on the examination of autopsy records, scientists have concluded that extracellular Aβ deposits (plaques) and intracellular neurofibrillary tangles (NFTs) are the two key pathological features associated with AD.

### 2.1 Aβ deposits

The brain contains a variety of Aβ deposits, also known as plaques. These include diffuse plaques, which are a mature form of Aβ deposits that appear in brain regions in the early stages of β-amyloidosis and have dense amyloid cores and neuroinflammatory pathology; cored neurologic plaques, which appear in the later stages of Aβ deposition and are a mature form of Aβ deposits that usually have dense Aβ cores; and neurologic plaques that have been associated with dementia (Ikonomovic et al., 2020).

#### 2.1.1 The formation of Aβ deposits

The APP protein is linked to Aβ deposits. Amyloid precursor protein (APP) is a transmembrane protein that has both cytoplasmic C-tails and soluble extracellular structural domains. There are 18 exons in the human APP gene, which is found on chromosome 21. The APP isoforms APP770, APP751, and APP695, as well as APLP1 and APLP2, are members of the human APP family (Cho et al., 2022; Galvão et al., 2019; O’Brien and Wong, 2011). APP affects a number of physiological processes, including blood coagulation regulation, cell adhesion, neuronal synapse formation, proliferation of neural stem or progenitor cells (NSPCs), and synaptogenesis (Dawkins and Small, 2014). Subsequently, two hydrolases, extracellular β-secretase and intracellular γ-secretase, cleave APP to generate Aβ (Esch et al., 1990; Galvão et al., 2019). When a mutation happens in APP, it results in elevated production of Aβ, which causes degenerative alterations in peripheral neurons and the development of age spots.

According to the amyloid cascade hypothesis, amyloid-β peptide (Aβ) is a key element in the pathophysiology of AD and is considered the primary cause of AD development (Hardy and Selkoe, 2002; Karran et al., 2011). Monomeric Aβ, a neuropeptide with 40–42 amino acids, is a physiological neuroprotectant whose in vivo concentrations are strongly linked to its physiological functions: (1) at picomolar concentrations, it regulates synaptic function, neural circuits, organelle transport, neurogenesis, neuroinflammation, and cognitive processes; (2) the Aβ monomer blocks microbial infections and stops the blood-brain barrier (BBB) from leaking at low concentrations (“vascular plug”); and (3) when present at physiological concentrations, Aβ safeguards the BBB, encourages brain damage healing, and suppresses tumor growth. In addition, Aβ monomers start to group together to produce unique Aβ oligomers, which subsequently form neuritic plaques (Devkota et al., 2021; Jeong et al., 2022; Kent et al., 2020; Mohamed Asik et al., 2021; Nichols et al., 2022; Penke et al., 2020).

#### 2.1.2 Predisposing factors for AD amyloidosis

The cause of AD amyloidosis is multifactorial. First, there is a disruption in cellular autophagy. In addition, autophagy is a comparable pathway for processing APP and APLP1 in neuronal cells under stress, as shown by Fangfang Zhou et al. Given the strong correlation between Aβ generation and APP processing, autophagy within neuronal cells could account for the accumulation of Aβ in AD (Zhou et al., 2011). ER stress and calcium dysregulation are the second most common causes. Since the ER is a crucial organelle for the control of calcium homeostasis, ER stress is typically linked to disruption of calcium homeostasis (Görlach et al., 2006). According to the findings of Eun Sun Jung et al., acute ER stress causes the development of chaperones associated with ERAD, which quickly breaks down APP via the ubiquitin-dependent proteasome pathway to eliminate APP that is misfolded or unfolded and stop the synthesis of Aβ. Conversely, APP buildup in the ER and increased Aβ synthesis are caused by proteasome malfunction induced by persistent ER stress (Jung et al., 2015). Third, inadequate cerebral blood flow can result in a lack of oxygen and energy in the brain, which can impact the synthesis of Aβ and its clearance mechanisms. Reduced cerebral blood flow is linked to the early identification of AD (Fazlollahi et al., 2020). Fourth, patients have elevated cholesterol levels. A protein found in the central nervous system called low-density lipoprotein receptor-related protein 1 (LRP1) aids in the removal of Aβ amyloidosis (Sagare et al., 2007). On the other hand, high cholesterol exacerbates apoptosis in the brain and causes cognitive impairment in AD mice by decreasing LRP1 expression, increasing the expression of the late glycosylation end product receptor RAGE, and increasing the expression of Aβ_40_ in brain microvascular endothelial cells. This results in deficient Aβ transport at the BBB (Zhou et al., 2021). Fifth, the acetylcholine dose is decreased. The key neurotransmitter that sustains consciousness is acetylcholine (Ach), and patients with AD exhibit a considerable decrease in Ach, which results in symptoms such as dementia. There are numerous possible explanations for the decreased acetylcholine concentration. For instance, Aβ_1–42_ directly induces a persistent increase in presynaptic Ca^2+^ levels and disturbs neuronal communication by blocking nicotine-mediated activation of the presynaptic nAChR, as discovered by John J. Dougherty’s team (Dougherty et al., 2003). However, one of the current therapeutic objectives for AD is the prevention of acetylcholinesterase oversynthesis, which successfully mitigates acetylcholine degradation (Rees and Brimijoin, 2003).

### 2.2 Neurofibrillary degeneration (NFT)

In neuroinflammatory (senile) plaques, neurofibrillary degeneration manifests as neurofibrillary tangles, nerve fiber bundles and dystrophic neuronal protrusions surrounding an amyloid core. Among the vast number of isolated tangles in the AD brain, the primary protein subunit of neuroprogenitor fiber tangles/paired helical filaments was identified by Western blot analysis as the microtubule-associated protein Tau (Grundke-Iqbal et al., 1986).

#### 2.2.1 Tau

Tau, a microtubule-associated protein (MAPT), is essential for the maintenance of complex neuronal cell microstructures, including microtubule assembly and stabilization, particularly in axons. It polymerizes microtubule proteins into microtubules (Avila et al., 2004). Microtubules and nerve cell plasma membranes are also susceptible to tau. Tau appears to influence synaptic function and encourage neuronal maturation through its presence in synapses and dendrites. Neuronal function can be impaired, and even AD can result from altered synaptic dispersion and disruption of the interaction of neurons with synaptic proteins. Among these pathways, one of the most illustrative pathogenetic pathways causing AD is tau hyperphosphorylation. In large filamentous tangles, Alonso AC et al. demonstrated that, in solution, normal tau binds nonsaturatingly to AD hyperphosphorylated tau (AD P-tau) to form large fine particles with a diameter of 3.3 ± 0.7 nm. In contrast, the ability of AD P-tau to aggregate with normal tau was eliminated by dephosphorylation with alkaline phosphatase, which also prevented the formation of tangles (Alonso et al., 1996). Bin Li and colleagues showed that separating normal brain tau and MAP2, an aberrantly hyperphosphorylated tau protein, from the cytoplasmic lysate of Alzheimer’s disease brains (AD P-tau) inhibits microtubule network construction and causes disruptions (Li et al., 2007). This implies that the hyperphosphorylation of Tau causes self-assembly of aggregates, segregation of normal MAPs, and disruption of microtubules, resulting in tangles of nerve fibres that impede normal nerve function and cause dementia symptoms.

#### 2.2.2 Tau hyperphosphorylation

Furthermore, the following are the variables that lead to Tau hyperphosphorylation: (1) An increasing number of studies validate the link between inflammation and Tau pathology in AD. According to Johanna Ojala et al., people with AD have higher amounts of IL-18 in their brains and CSF. IL-18 is found in neurons as well as blood vessels and neuroglia linked to Aβ plaques. IL-18 could play a significant role in the neuroinflammatory response vicious cycle that AD sufferers’ brains experience (Ojala et al., 2009). Christina Ising et al. also showed that loss of NLRP3 inflammatory vesicle function reduces tau hyperphosphorylation and aggregation by regulating tau kinase and phosphatase, that Tau activates NLRP3 inflammatory vesicles and that intracerebral injection of fibrillar β-amyloid-containing brain homogenate induces tau pathology in an NLRP3-dependent manner (Ising et al., 2019). (2) Another significant factor in Tau hyperphosphorylation is the gene. The most potent genetic risk factor for AD is APOE4, which has been shown to increase brain amyloid-β plaques more than other ApoE isoforms. However, Yang Shi et al. showed that the brains of P301S/E4 mice had significantly greater levels of tau protein and that P301S/E4 P301S/E4 mice had significantly greater brain atrophy and neuroinflammation than did P301S/E2 and P301S/E3 mice. In contrast, P301S/EKO mice were largely protected from these changes, indicating that ApoE influences tau pathogenesis, neuroinflammation, and tau-mediated neurodegeneration independently of Aβ pathology. While an absence of ApoE is protective against tau-mediated neurodegeneration, ApoE4 plays a “toxic” role in the process of tau accumulation (Shi et al., 2017). (3) Additionally, through dysregulation or in certain ways, protein kinases and phosphatases can hyperphosphorylate Tau. Among these pathways, the PI3K/AKT/GSK-3β pathway is a crucial pathway that induces Tau hyperphosphorylation in AD; in addition, aberrant Tau hyperphosphorylation is linked to glycogen synthase kinase-3β (GSK-3β), cell cycle protein-dependent kinase 5 (CDK5), and p38 mitogen-activated protein kinase (p38-MAPK)(Hu et al., 2013; Wu et al., 2008). (4) Tau phosphorylation may result from specific medical conditions, therapeutic regimens, or prescribed drugs. It is increasingly recognized that cognitive dysfunction is a significant comorbidity of diabetes. Over the past ten years, there has been a convergence of evidence from brain autopsy studies suggesting that persons with type 2 diabetes mellitus (T2DM) and those without this disease do not exhibit any greater prevalence of the basic neuropathologic characteristics of AD (Arvanitakis et al., 2006). The main characteristic of insulin resistance is interference with insulin signaling, which results in insulin resistance. Insulin resistance impacts systemic metabolism and the brain directly by interfering with the brain’s insulin pathway (Arnold et al., 2018).

## 3 Directions for AD treatment and the current status of clinical trials

Several variables are involved in the pathogenesis of Alzheimer’s disease, and these targets have been extensively studied by researchers worldwide. Currently, donepezil and memantine are FDA-approved medications for the treatment of AD. However, these medications are limited to treating mild and moderate AD symptoms, and to have any therapeutic benefit, they must be given as soon as AD symptoms start. Currently, there are no FDA-approved medications that target core pathologies or provide long-term treatment options for advanced AD patients. Numerous medications have been tested in clinical trials, but they have all failed at different points and have shown a number of negative side effects. The clinical studies that have been conducted in recent years to treat Aβ amyloid deposition plaques and tau-related neurofibrillary tangles are included in Tables 2, 3.

**TABLE 2:** Treatments based on Aβ plaques.

Drug	Therapeutic mechanism	Findings	Adverse events	ClinicalTrials.gov number	References
Lecanemab (BAN240)	Humanized monoclonal antibody that binds with high affinity to soluble amyloid-beta (Aβ) protofibrils	Mop up beta amyloid and it modestly slowed cognitive decline	Brain swelling (ARIA-E for edema) and brain bleeding (ARIA-H for hemorrhage) infusion-related reactions, ARIA-E, atrial fibrillation, syncope and angina pectoris	NCT03887455	van Dyck et al., 2023
Donanemab	Humanized IgG1 antibody directed at an N-terminal pyroglutamate Aβ epitope	Reduced the amyloid plaque level and slowed cognitive decline	Amyloid-related cerebral edema or effusions occurred with donanemab ARIA-E, confusion, difficulty with expressing, superficial siderosis of the central nervous system, nausea, infusion-related reactions, Antidrug antibodies were detected	NCT03367403	Mintun et al., 2021
HTL9936	Lead M1-receptoragonist that would activate receptors in the CNS in areas such as the cortex and hippocampus involved in learning and memory	Exhibits pro-cognitive effects in animal models of Alzheimer’s disease and activates brain regions associated with learning and memory in humans.	Salivation, sweating, and changes in blood pressure and/or heart rate	NCT02291783	Brown et al., 2021
AL002c	A mouse anti-hTREM2 agonistic mAb named AL002c	mAb-mediated engagement of hTREM2 enhancesneuroprotective microglia, restraining neurotoxic Aβ plaques and neuronal damage	/	NCT03635047	Wang et al., 2020
Solanezumab	Humanized immunoglobulin G1 monoclonal antibody that binds to the mid-domain of the Aβ peptide	Increase the clearance from the brain of soluble Aβ, peptides	Vitamin D deficiency, nasal congestion, spinal osteoarthritis, and dysuria;gait disturbance and somnolence, Antidrug antibodies occurred, edema or effusions on cerebral imaging	NCT01900665	Honig et al., 2018
CNP520	The BACE-1 inhibitor CNP520	CNP520 reduced brain andcerebrospinal fluid (CSF) Ab in rats and dogs, and Ab plaque deposition in APP-transgenic mice	Generalized pruritus of moderate severity, AE of transient global amnesia	NCT02565511 and NCT03131453	Neumann et al., 2018
Donepezil (Aricept™), rivastigmine (Exelon™), galantamine (Razadyne™)	Acetylcholinesterase inhibitors (AChEI)	Restoration of defective cholinergic transmission via inhibition of the cholinesterases	Cardiovascular(Fast or slowed heart rate, Irregular heartbeat (arrhythmia))Neuro (Fainting, Dizziness, Nightmares, Headache, Seizures or fits, Sleeplessness or poor sleep (insomnia)) Gastrointestina(Upset stomach, Vomiting, Diarrhea, Lack of interest in eating (anorexia))Musculoskeletal(Muscle cramps, Pain, Falls, Feeling tired (Fatigue), Tremor or shaking), Incontinence of urine or stool, Asthma/wheezing or trouble breathing, Runny nose (rhinitis)	NCT01362686	Campbell et al., 2017
Riluzole	A glutamate modulator that exhibits neuroprotective properties	Preventing further damage to vulnerable pyramidal neurons through modulation of glutamate levels, reduced glutamate-mediated toxicity and increased synaptic activity. Riluzole has shown similar mitigating effects on sodium and calcium ion influx, slows cerebral glucose metabolism decline	Diarrhea, dizziness, urinary frequency, nausea, cough, elevated liver enzymes	NCT01703117	Matthews et al., 2021
Atabecestat(JNJ-54861911)	A potent brain-penetrant BACE inhibitor	JNJ-54861911 at 10 and 50 mg daily doses after 4 weeks resulted in mean CSF Aβ_1–40_ reductions of 67%and up to 90% in both Caucasian and Japanese patients with early stage AD, confirming results in healthy elderly adults.	Psychiatric disorders, Anxiety, Depressed mood, Insomnia, Irritability, Renal colic, Vulvovaginal pruritus, Dermatitis allergic, Eczema vesicular, Psoriasis, Urticaria, Vascular disorders, Hypertension, Subjects with any TEAE, Fatigue, Infections and infestations, Conjunctivitis, Nasopharyngitis, Vulvovaginal candidiasis, Injury, poisoning and procedural complications, Accidental overdose, Post lumbar puncture syndrome, Musculoskeletal and connective tissue disorders, Neck pain, Nervous system disorders, Dementia, Headache	NCT01978548 and NCT02360657	Timmers et al., 2018

**TABLE 3:** Clinical studies involving Tau and NFTs.

Drug	Therapeutic mechanism	Findings	Adverse events	ClinicalTrials.gov number	References
Bapineuzumab	A humanized N-terminal–specific anti-Aβ monoclonal antibody	Had a greater reduction in amyloid on positron-emission tomographicamyloid imaging and reduced cerebrospinal fluid phosphorylated tau (phospho-tau), suggesting target engagement andattenuated neurodegeneration	Serious TEAE, Nervous system disorders, Neoplasms benign, malignant and unspecified (incl cysts and polyps), Injury, poisoning and procedural complications, Psychiatric disorders, Infections and infestations, Gastrointestinal disorders, Respiratory, thoracic and mediastinal disorders, Metabolism and nutrition disorders, Musculoskeletal and connective tissue disorders, Hepatobiliary disorders, Vascular disorders, Renal and urinary disorders, General disorders and administration site conditions, Investigations, Reproductive system and breast disorders, Blood and lymphatic system disorders, Ear and labyrinth disorders, Immune system disorders, Congenital, familial and genetic disorders, Skin and subcutaneous tissue disorders	NCT00575055 and NCT00574132	Salloway et al., 2014
Semorinemab(previously known as RO7105705,MTAU9937A, or RG6100)	A humanized IgG4 monoclonal antibody that targets the N-terminal domain of tau	Semorinemab failed to demonstrate meaningful efficacy on clinical end points or reduction of tau accumulation across 18months in participants with prodromal to mild AD at the doses administered.	Fall, Nasopharyngitis, Infusion-related reaction, Arthralgia, Hypertension, Urinary tract infection, Upper respiratory tract infection, Back pain, Dizziness, Anxiety, Diarrhea, Headache, Nausea, Cough, Depression, Agitation, Insomnia, Blood pressure increased, Fatigue, Skin laceration	NCT03289143	Teng et al., 2022
Crenezumab	A humanized anti-Aβ monoclonal immunoglobulin G4 antibody	Crenezumab was well tolerated but did not reduce clinical decline in participants with early AD	Headache, Nasopharyngitis, Fall, Hypertension, Back pain, Upper respiratory tract infection, Anxiety, Depression, Diarrhea, Dizziness, Pneumonia, Subdural hematoma, Syncope, Dehydration, edema, hemorrhage, Infusion-related reactionsd	CREAD, NCT02670083; CREAD2,NCT03114657	Ostrowitzki et al., 2022
MAPTRx (ISIS 814907/BIIB080)	Antisense oligonucleotide (ASO)	MAPTRx prevents tau protein production, and should lower the levels of all tau species and subsequent posttranslational modifications with the potential to reduce both pathological spreading of extracellular tau and neuronal dysfunction due to intracellular tau accumulation	Post-LP headache, Procedural pain, Musculoskeletal pain, Vomiting, Back pain, Confusional state, Contusion, Diarrhea, Dizziness, Fatigue, Myalgia, Nasopharyngitis, Nausea, Tinnitus	NCT03186989	Mummery et al., 2023
leuco-methylthioninium bis[hydromethanesulfonate]; LMTM	tau-aggregation inhibitor	The primary analysis for this study was negative, and the results do not suggest benefit of LMTM as an add-on treatment for patients with mild to moderate Alzheimer’s disease	Blood and lymphatic system disorders, Anaemia, Gastrointestinal disorders, Diarrhoea, Nausea, Vomiting, Abdominal pain, Infections and infestations, Urinary tract infection, Blood folate decreased, Weight decreased, Haemoglobin decreased, Metabolism and nutrition disorders, Folate deficiency, Nervous system disorders, Cognitive disorder, Renal and urinary disorders, Dysuria, Micturition urgency, Pollakiuria, Urinary incontinence, Respiratory, thoracic, and mediastinal disorders, Cough	NCT01689246	Gauthier et al., 2016
AADvac1	An active peptide vaccine	AADvac1 can induce a selective, pathological, tau-specifi c antibody response in elderly patients. The cognition of treated patients remained stable throughout the 6 months.	Injection site erythema, Injection site swelling, Injection site warmth, Injection site pain, Back pain, Arthralgia, Urinary tract bacterial infection, Haematuria, Hypertension	NCT01850238 and NCT02031198	Novak et al., 2017
Tideglusib	A thiadiazolidinone which irreversibly inhibits GSK-3	It did not show clinical efficacy in mild to moderate AD patients enrolled into the ARGO study.	Diarrhea, ALT increased, Nasopharyngitis, Nausea, Vomiting, Urinary tract infection, AST increased, GGT increased, Headache, Cough, Flatulence, Syncope	NCT01350362	Lovestone et al., 2015

The two main treatments being tested in clinical trials for Aβ amyloid deposition plaques are small molecule inhibitors and immunotherapy. Among these, small molecule inhibitors primarily focus on inhibiting β- and γ-secretases. A recent announcement regarding the drug’s withdrawal from the phase III trial revealed that atabecestat, also referred to as JNJ-54861911, a BACE inhibitor, decreased CSF Aβ_1–40_ in patients with early AD after 4 weeks of dosing. Additionally, the drug has been shown to cause a variety of adverse effects, primarily elevated liver enzymes (Timmers et al., 2018). And the newer generation of “γ-secretase allosteric stabilizers” (GSAS) are being tested. The prototype, introduced in 2006 by Eisai (Yu et al., 2014), targets an allosteric site in the PSEN subunit (Cai et al., 2017; Yang et al., 2021), which are stabilizers of the enzyme that enhances APP processing in affected neurons (De Strooper and Karran, 2024). Additionally, acetylcholinesterase inhibitors (AChEIs) are utilized to extend cholinergic neurotransmission and signaling in patients with symptomatic AD, but the study population has also experienced adverse effects in the CNS and musculoskeletal system (Bond et al., 2012; Campbell et al., 2017). Among the most promising immunotherapies, however, are humanized monoclonal antibodies such as Lecanemab (BAN2401), which bind highly affinity to soluble amyloid-β (Aβ), eliminating β-amyloid and only slightly slowing cognitive decline in the early stages of the disease. Despite being approved, however, research on potentially severe brain hemorrhage and swelling is still lacking (Couzin-Frankel, 2023; Honig et al., 2018; Mintun et al., 2021; van Dyck et al., 2023).

Analogous to Aβ immunotherapy, the humanized IgG4 monoclonal antibody semorinemab has been developed to block Tau aggregation, but it has not been found to decrease or clinically slow the rate of cerebral tau accumulation in prodromal patients with mild Alzheimer’s disease (Teng et al., 2022). In contrast, AADvac1, an active peptide vaccine, generates IgG antibodies that can target tau microtubule site epitopes, decrease neuroprogenitor fibre tangles and ameliorate deleterious phenotypes in Tau patients, and in older patients, it causes a pathologic, selective, tau-specific antibody response (Novak et al., 2017). The microtubule-associated protein tau (MAPT) gene encodes the tau protein. MAPTRx (ISIS 814907/BIIB080) is an antisense oligodeoxynucleotide (ASO) intended to lower the mRNA concentration of MAPT and consequently the protein level of tau. However, 94% of subjects treated with MAPT experienced adverse events, the most common of which was headache following lumbar puncture (LP)(Mummery et al., 2023). An analog of thiadiazolidinone, tideglusib, inhibits GSK-3 irreversiblely. In multiple laboratory models, tideglusib has been demonstrated to decrease tau hyperphosphorylation, Aβ deposition, neuronal loss, and inflammatory and neurotrophic deficiency markers; however, in ARGO research, tideglusib did not demonstrate any therapeutic efficacy in patients with mild to moderate AD (Lovestone et al., 2015).

The most common therapeutic approach to stop, slow, or even reverse the progression of AD pathology has been to decrease tau or Aβ levels. In clinical trials, however, they are experiencing trouble because the effects do not seem to be correlated with improved symptoms. It is believed that the problem with anti-Aβ amyloid deposition monoclonal antibody-based treatments is that they are being used when the disease is already very advanced. That is why they are currently being tested in young people carrying familial variants in *PSEN1*, *PSEN2* and *APP* to study if they can delay or prevent the development of the disease (Liu et al., 2024; Wagemann et al., 2024). At the same time, early markers of the disease are being sought that allow patients to be diagnosed before cognitive deterioration, which could improve the effectiveness of treatments. Ubiquitin-Proteasome System in the Different Stages of Dominantly In review Inherited Alzheimer’s Disease (DIAD) have been noticed by Eric McDade, and a positive association was observed with imaging markers (PiB PET, tau PET) and a negative correlation with markers of neurodegeneration (FDG PET, MRI), highlighting a significant link between UPS dysregulation and neurodegenerative processes, implicating the UPS as a potential therapeutic target in AD pathogenesis (McDade et al., 2024). There are more markers for the early diagnosis of Alzheimer’s Disease, such as Osteopontin, apathy and anxiety, and the crosstalk Between Amyloid-β, retina, and sleep, contributing to the treatment of Alzheimer’s disease (De Guia et al., 2024; Johansson et al., 2020; Quesnel et al., 2024).

## 4 Stem cells in animal models of AD

Currently, FDA-approved medications have substantial adverse effects, can only be used to treat symptoms, and cannot fully cure AD. Approaches to treating AD have been revolutionized by stem cell therapy, which aims to restore damaged neurons. With stem cell therapy emerging as a promising treatment option for AD, stem cell technology is becoming increasingly sophisticated and has already shown promise in preclinical tests. Embryonic stem cells (ESCs), mesenchymal stem cells (MSCs), brain-derived neural stem cells (NSCs), and induced pluripotent stem cells (IPSCs) are the cells most frequently employed in AD research, and we also conclude in Table 4.

**TABLE 4:** Models of stem cell therapy for AD.

Cell type	Therapeutic mechanism	Findings	Route of administration	Model	References
hUCB-MSCs	Recovery of NEP expression to increase Aβ clearance, NEP was induced by coculturing hUCB-MSCs in microglia cells.	transplantation of hUCB-MSCs improved spatial memory deficits and reduced Aβ deposits in a transgenic mouse model of AD	hUCB-MSCs were stereotactically inoculated into the hippocampal area or the cisterna magna	Double transgenic mice, Mo/Hu APPswe PS1dE9	Kim et al., 2012
Protein-iPSC	Remain unknow.	5XFAD mice treated with protein-iPSCs exhibited decreased plaque deposition, but transplanted protein-iPSCs did not differentiate into neuronal cells	stereotaxic injection in the subiculum area	5XFAD mice	Cha et al., 2017
human adult ischemia-tolerant mesenchymal stem cells (hMSCs) and factors (stem cell factors)	a beneficial impact of hMSC through upregulation of the levels of certain Aβ-degrading enzymes leading to increased Aβ clearance.	A single hMSC injection markedly reduced soluble cerebral Aβ levels in APPPS1 mice after 1 week,10 weeks of hMSC treatment significantly reduced cerebral Aβ plaques and neuroinflammation, a repeated intranasal delivery of soluble factors secreted by hMSCs in culture, in the absence of intravenous hMSC injection, was also sufficient to diminish cerebral amyloidosis in the mice.	hMSCs were administered intravenously and a repeated intranasal delivery of soluble factors secreted by hMSCs in culture	APPPS1 transgenic mice	Harach et al., 2017
human induced pluripotent stem (iPS) cell-derived macrophage-like cells, iPS-ML	the reduction of Aβ was caused by the secretion of NEP2 from the iPS-ML, but not phagocytosis of Aβ by the iPS-ML	transplantation of iPS-ML/NEP2 significantly diminished the levels of soluble Aβ_1–42_ in the brain ISF compared to the control Ringer’s solution injection	stereotaxically inserted microinjection tubes into the hippocampus of 5XFAD mice and transplanted iPS-ML through this tube.	5XFAD mice	Takamatsu et al., 2014
human ESC-derived BFCNs	ESCderived BFCNs replacement and partial reconstruction of damaged cholinergic neuron circuitry of AD model mice	both mouse and human ESCs efficiently differentiated into mature and functional BFCNs and these ESC-derived BFCNs integrated into the basal forebrain of AD mice and alleviated the cognitive deficits of AD.	The bilateral transplantation was performed by the injection	Transgenic AD model mice: 5XFAD and APP/PS1.	Yue et al., 2015
Exosomes derived from bone-marrow mesenchymal stem cells (BMSC-exos)	the mechanism might be involved in the regulation of glial activation and its associated neuroinflammation and BDNF-related neuropathological changes in the hippocampus.	lateral ventricle administration of BMSC-exos could improve ADlike behaviors, as indicated by the increased motion and rearing in the OFT, together with an increased preference index of novel objects in the NOR. Moreover, hippocampal inflammation and hyperactivation of glial cells in the AD mouse model were significantly improved, and the protein expression levels of BACE, Aβ_1–42_ and p-Tau were significantly reduced, while the expression level of BDNF was significantly increased. However, not caudal vein injection of BMSC-exos, can improve AD-like behaviors in	BMSC-exos were administered via lateral ventricle injection or caudal vein injection	A sporadic AD mouse model was established by intracerebroventricular injection of streptozotocin (STZ)	Liu S. et al., 2022
Human neural stem cells	The hNSC transplantation reduced tau phosphorylation via Trk-dependent Akt/GSK3β signaling, downregulated Aβ production through an Akt/GSK3β signaling-mediated decrease in BACE1, and decreased expression of inflammatory mediators through deactivation of microglia that was mediated by cell-to-cell contact, secretion of anti-inflammatory factors generated from hNSCs, or both.	The hNSC transplantation improved spatial memory in these mice, which also showed decreased tau phosphorylation and Aβ_42_ levels and attenuated microgliosis and astrogliosis.	intracerebroventricular transplantation	neuron-specific enolase promoter-controlled APPsw-expressing (NSE/APPsw) transgenic mice	Lee et al., 2015
Neural stem cell (NSC)	cell transplantation may enhance ChAT protein levels and restore cholinergic neurons.	NSC transplantation can protect basal forebrain cholinergic neurons and restore synaptic impairment as well as eventually improve learning and memory functions in an APP/PS1 transgenic (Tg) mouse model.	Surgical transplantation of NSCs into the hippocampus.	APP/PS1 transgenic mice	Zhu et al., 2020
exosomes from hypoxic pretreated adipose-derived stem cells (ADSCs)	hypoxic pretreatment of ADSC exosomes improved cognition by delivery of circEpc1 and by shifting microglial M1/M2 polarization in an AD mouse model.	exosomes from hypoxia pretreated ADSCs had a good therapeutic effect on improving cognitive functions by decreasing neuronal damage in the hippocampus. circ-Epc1-containing ADSC exosomes increased the therapeutic effect of exosomes by improving cognitive function, decreasing neuronal damage, and shifting hippocampal microglia from the M1 polarization to the M2 polarization stages.	injections	APP/PS1 double transgenic AD model mice	Liu H. et al., 2022
human neural stem cell (hNSC)-derived EVs(extracellular vesicles)	the neuroprotective effects of EVs are exerted, in part, by reducing the plaque burden in the AD brain at early and late stages of disease progression. Dysfunction of neural circuitry in the hippocampus, mPFC, and amygdala that could be ameliorated by EV treatment. EV treatment is affecting multiple pathways to dampen inflammation. hNSC-derived EVs may repair the AD brain, delivering miRNA capable of reducing inflammation and protecting neuronal structure, possibly through epigenetic regulation of gene expression.	RO treatment using hNSC-derived EVs restored fear extinction memory consolidation and reduced anxietyrelated behaviors 4–6 weeks postinjection. EV treatment also significantly reduced dense core amyloid-beta plaque accumulation and microglial activation in both age groups.	intravenous (retro-orbital vein, RO) injections	the 5xFAD accelerated transgenic mouse model of AD	Apodaca et al., 2021

### 4.1 ESCs

With the exception of the placenta, pluripotent stem cells called embryonic stem cells (ESCs) can be grown in vitro and still differentiate into each of the three embryonic germ layers (Conley et al., 2004). Embryonic stem cells can differentiate into basal forebrain cholinergic neurons or BFCNs. Following the transplantation of BFCNs generated from mouse and human ESCs into the basal forebrain of AD model mice, Wei Yue et al. reported that animals with AD exhibited enhanced learning and memory performance when BFCN progenitors mostly developed into mature cholinergic neurons. These neurons then functionally integrate into the endogenous basal forebrain cholinergic projection system, alleviating cognitive impairments in AD animals (Yue et al., 2015). As above, Yan Liu et al. differentiated human embryonic stem cells (hESCs) into a nearly homogenous population of NKX2.1 MGE-like progenitor cells. These progenitor cells give rise to GABA interneurons and BFCNs, which are connected with host hippocampal neurons to rectify learning and memory deficits. The mice with medial septal lesions were then transplanted with these MGE-like progenitor cells (Liu et al., 2013). Although there are many data supporting the pluripotency of ESCs, there are inherent therapeutic risks involved, in which their clinical application their is limited by immunogenic rejection, unregulated cell proliferation, and cancer-causing potential (Lee et al., 2013). At present, one of the main areas of research is the use of ESCs to create animal disease models (Honda et al., 2016; Kim et al., 2020; Mancuso et al., 2019).

### 4.2 MSCs

MSCs have the capacity to differentiate into mesodermal cells, which include adipocytes, chondrocytes, and osteoblasts. MSCs and neural cells may interact, primarily through bystander processes. MSCs can produce a neuroprotective milieu by exerting anti-inflammatory and antiproliferative effects on microglia and astrocytes and rescue neurons and oligodendrocytes from apoptosis by releasing trophic and antiapoptotic chemicals. Furthermore, MSCs can stimulate the growth and development of localized neural precursor cells, allowing them to differentiate into adult neurons and oligodendrocytes (Uccelli et al., 2008). Several studies have even demonstrated that MSCs may have immunomodulatory effects and certain suppressive effects on T-cell proliferation. These properties could aid in preventing immunological rejection following MSC transplantation (Ankrum et al., 2014; Bartholomew et al., 2002; Di Nicola et al., 2002).

MSC-based treatments for AD have also demonstrated some success in preclinical research. APPPS1 transgenic mice were given intravenous injections of hMSCs by Taoufiq Harach et al. These findings indicate that hMSCs have amyloid-degrading and anti-inflammatory properties. A single injection of hMSCs significantly reduced soluble brain Aβ levels in APPPS1 mice after one week, and a 10-week treatment with hMSCs significantly reduced the number of brain Aβ plaques and neuroinflammation in APPPS1 mice without increasing cerebral amyloid angiopathy or microhemorrhages (Harach et al., 2017). Furthermore, the significance of stem cell-derived exosomes has been steadily highlighted in recent research. BMSC-exosomes (produced from bone marrow mesenchymal stem cells) carry out the functions of BMSCs. Sen Liu et colleagues. found that injecting BMSC-exos via the lateral ventricle significantly reduced hippocampal inflammation and neuroglial overactivation in an AD animal model and improved AD-like behavioral performance in mice. Significant decreases in the protein expression levels of BACE, Aβ_1–42_, and p-Tau were observed, while significant increases in BDNF expression were observed (Liu S. et al., 2022).

### 4.3 NSCs

Neural stem cells are also multipotent stem cells that are mostly present in the developing and central nervous systems of mammals. They can differentiate into neurons, astrocytes, and oligodendrocytes (Gincberg et al., 2012). In AD therapy, neural stem cells (NSCs) function primarily through two mechanisms. First, damaged neurons are repaired through direct transplantation. The other group controls brain synaptic activity by secreting a range of neurotrophic factors, which are mediated via paracrine actions.

An AD amyloid precursor protein (APP)/progerin 1 (PS1) transgenic (Tg) mouse model was generated by seeding human brain-derived neural stem cells (hNSCs) by Xueyuan Li et al. In AD mice, hNSC transplantation corrected impairments in identification, learning, and memory but not in anxiety tests. Aβ plaques did not significantly decrease, but the densities of neurons, synapses, and neurofibrils in the frontal cortex and hippocampus of hNSC-treated AD mice significantly increased (Li et al., 2016). Similarly, Qing Zhu et al. reported increased expression of the protein choline acetyltransferase (ChAT) and restoration of cholinergic neurons in the basal forebrain following NSC transplantation. Furthermore, the expression of synaptophysin (SYP), postsynaptic density protein 95 (PSD-95), and microtubule-associated protein 2 (MAP-2) was considerably upregulated in the hippocampal regions of AD mice treated with NSCs, and spatial learning and memory were enhanced (Zhu et al., 2020). With the guidance of the rat neuron-specific enolase (NSE) promoter, Il-Shin Lee et al. investigated the potential therapeutic benefits of human fetal brain-derived NSCs in NSE/APPsw transgenic mice harboring a Swedish mutant of APP. They discovered that the implanted cells migrated and engrafted extensively and that some of the cells differentiated into neurons and glial cells, which improved the spatial memory of the mice. Trk-dependent Akt/GSK3β signaling decreased tau phosphorylation, downregulated Aβ production through the Akt/GSK3β signaling-mediated reduction in BACE1, and decreased the expression of inflammatory mediators through microglia-mediated inactivation or secretion of anti-inflammatory factors generated by hNSCs or both. By providing nutrients and acting as an antiapoptotic agent, hNSCs also enhance synaptic plasticity (Lee et al., 2015).

### 4.4 iPSCs

In 2006, Yamanaka published a groundbreaking study on the reprogramming of cells to become pluripotent stem cells from mouse embryonic or adult fibroblasts by introducing four factors: Oct3/4, Sox2, c-Myc, and Klf4. This process is termed “induced pluripotent stem” (iPS) and was carried out under ESC culture conditions (Takahashi and Yamanaka, 2006). This work was awarded the 2012 Nobel Prize in Medicine together with Sir John Gurdon’s previous study on reprogramming utilizing somatic cell nuclear transplantation (Abbott, 2012). The practical differentiation capability of iPS cells is believed to be equivalent to that of ESC, and iPSC acquisition minimizes some immunogenic and ethical issues.

Researchers Moon-Yong Cha et al. discovered that transplanting Protein-iPSCs into a 5XFAD transgenic AD mouse model resulted in glial cell differentiation, decreased plaque formation, and decreased cognitive impairment (Cha et al., 2017). On the other hand, Edsel M. Abud’s group discovered that microglia-like cells (iMGLs) that were differentiated from iPSCs grow in vitro similarly to microglia in vivo. Their functional evaluation revealed that these cells migrate through calcium transients, strongly phagocytose CNS substrates, and secrete cytokines in response to inflammatory stimuli. When iMGLs are implanted into human brain organoids and transgenic mice in vivo, the iMGLs resemble microglia (Abud et al., 2017). Enkephalinase-2 (NEP2), a protease with Aβ-degrading activity, was expressed by Koutaro Takamatsu et al. in iPS-ML, a macrophage-like myeloid cell of iPS cell origin. In vitro, NEP2 expression enhanced the reduction in the level of soluble Aβ oligomers in the culture medium and attenuated Aβ neurotoxicity. Since intracerebral delivery of iPS-ML/NEP2 to 5XFAD mice resulted in a considerable decrease in Aβ levels in the brain interstitial fluid, these findings imply that iPS-ML/NEP2 may be a viable therapeutic agent for the treatment of AD (Takamatsu et al., 2014).

## 5 Clinical trials of stem cells for AD treatment

When Alzheimer’s disease and stem cells were entered into ClinicalTrials.gov, a search of seven completed clinical studies was performed, as shown in Table 5.

**TABLE 5:** Stem cells in clinical trials for AD.

ClinicalTrials.gov number	Interventions	Route of administration	Findings	First Posted and Stage	Conclusion	References
NCT01297218 and NCT03172117	Biological: Human Umbilical Cord Blood Derived-Mesenchymal Stem Cells	Stereotactic injection	No patient showed serious adverse events including fever during the 24-month follow-up period. During the 12-week follow-up period, the most common acute adverse event was wound pain from the surgical procedure. There was no dose-limiting toxicity.	2011/2/11, Phase 1 Clinical Trial to Evaluate the Safety and the Efficacy	Administration of hUCB-MSCs into the hippocampus and precuneus by stereotactic injection was feasible, safe, and well tolerated. Further trials are warranted to test the efficacy.	Kim et al., 2015
NCT02600130	Biological: Longeveron Mesenchymal Stem CellsBiological: Placebo	Hippocampal volume. Ventricular volume. Whole-brain volume.	Not provided	2015/11/9, A Phase 1 Trial to Evaluate the Safety and Potential Efficacy	Not provided	/
NCT03117738	Drug: AstroStemOther: Placebo-Control	administered via I.V.	Not provided	2017/4/18, Phase 1/2, to Evaluate the Safety and Efficacy	Not provided	/
NCT04040348	Biological: Approximately 100 million cells allogeneic hMSC	administered intravenously	Not provided	2019/7/30, Phase 1 Trial to Evaluate the Safety	Not provided	/
NCT04052737	Drug: Normal Saline along with standard treatmentDrug: PMZ-1620 (sovateltide) along with standard treatment	administered as an IV bolus	Intravenous administration of PMZ-1620 (sovateltide) augments the activity of neuronal progenitor cells in the brain to repair the damage by formation of new mature neurons and blood vessels. In addition, PMZ-1620 has anti-apoptotic activity and increases cerebral blood flow.	2019/8/8, Phase II Clinical Study to Compare the Safety and Efficacy	A dose of 0.6 microg/kg of IRL-1620 can be safely administered, three times every four hours, without any adverse effect.	Briyal et al., 2014; Briyal et al., 2015; Gulati et al., 2018

The majority of their research was in Phase 1 or Phase 2, with the main objective being to assess the effectiveness and safety of stem cell preparations. For instance, Hee Jin Kim et al. conducted a phase 1 clinical study (NCT01297218) in Korea to assess the safety and dose-limiting toxicity of stereotactic intracerebral injection of mesenchymal stem cells from human umbilical cord blood (hUCB-MSCs) (Kim et al., 2015). Additionally, none of the patients experienced serious adverse events, such as fever, during the 24-month follow-up period, and during the 12-week follow-up period, the most common acute adverse event was only wound pain from surgery (n = 59), with no toxicity that exceeded a certain dose. These results demonstrated that stereotactic injection of hUCB-MSCs into the hippocampus and precuneus is safe, feasible, and well tolerated (Kim et al., 2015). Hee Jin Kim et al. subsequently assessed the safety and dose-limiting toxicity of three ventricular injections of human umbilical cord blood mesenchymal stem cells (hUCB-MSCs) in the context of NCT03172117 (Kim et al., 2021). Four weeks before the patients received MSC treatment, Ommaya reservoirs were inserted into their right ventricles. Three MSC injections were administered to each patient, which were separated by four weeks. Following their initial hUCB-MSC injection, these patients were monitored for up to 12 weeks and then for an additional 36 months as part of an extended observational study. Three repeated injections of hUCB-MSCs into the lateral ventricle via the Ommaya reservoir appear to be feasible, relatively safe, and well tolerated. Fever (*n* = 9) was the most common adverse reaction following hUCB-MSC injection, and five participants completed the 36-month extended observational study without experiencing any additional serious adverse events (Kim et al., 2021). A phase II clinical trial by Briyal et al. also compared the safety and effectiveness of treating mild to moderate Alzheimer’s disease patients with PMZ-1620 (Briyal et al., 2014). As an agonist of the endothelin B (ETB) receptor, PMZ-1620 promotes neurovascular remodeling and repair as well as nerve regeneration when it binds to the ETB receptor. After brain injury, dormant stem cells in the brain become activated. When PMZ-1620 (sauvatide) is injected intravenously, it activates the brain’s neural progenitor cells, which create new, mature neurons and blood vessels to heal the damage. PMZ-1620 also has antiapoptotic effects and enhances blood flow to the brain. The findings in NCT04052737 demonstrated that IRL-1620 can be taken three times every four hours at a dose of 0.6 μg/kg without the risk of side effects (Briyal et al., 2014; Briyal et al., 2015; Gulati et al., 2018).

In addition, other clinical trials involving stem cell therapy for Alzheimer’s disease are currently being conducted. The first is a phase IIa study led by Stemedica Cell Technologies, which aimed to assess the safety and tolerability of ischemia-tolerant allogeneic human mesenchymal stem cells (hMSCs) administered intravenously to individuals with mild-to-moderate dementia induced by the disease. The participants were divided into two groups of 20, with each group randomly assigned a 1:1 ratio to receive either the active study drug or a placebo, and the participants were scheduled to conclude on December 31, 2024. The second trial is a phase 1 open-label safety study in which Regenerative BioMedicine, Inc., started to investigate the impact of increasing the volume of intracerebral injections of this investigational product on the brain volume of in vitro-expanded autologous adipose-derived stem cells (ADSCs) in individuals with mild-to-moderate Alzheimer’s disease (ADS). The experimental product, called RB-ADSC, is made of stem cells that were extracted through liposuction from the adipose tissue of participants. Following their extraction, the stem cells were grown and cultivated in a laboratory setting before being reinfused into the original patient. Under the scalp, an Ommaya reservoir—a soft plastic reservoir attached to the ventricle of the brain—was inserted. When determining the recommended Phase 2 clinical trial dose, the main goal will be to assess major adverse events, and dose-limiting toxicities. This trial is anticipated to be completed by 2025–03.

Besides, the Development of iPS From Donated Somatic Cells of Patients With Neurological Diseases (NCT00874783) is active, but not recruiting. The major goal of the project is to develop human iPS cells from cell cultures from skin biopsies or the patient’s hair. The iPS cells will be developed primarily for modeling diseases and drug discovery as well as basic research, and for developing the technology that may eventually allow the use of iPS cells for future transplantation therapy. And the Rajavtihi Neuronal Adult Stem Cells Project (RNASc)(NCT00927108) was withdrawn, with the reason remains unknown.

## 6 Discussion and perspective

Additional studies are needed to demonstrate the safety and effectiveness of stem cell therapy in humans, despite the large number of pertinent preclinical trials that have been conducted. Why is there still poor translation between animal research and human trials in stem cell therapy for AD? What challenges does the development of stem cell treatment currently face, and how may these challenges be overcome? Currently, experts are interested in how they may be successfully used for diseases that are difficult to cure, such as AD.

### 6.1 Safety and immaturity of stem cell technology

Pharmaceutical drugs derived from stem cells have potential unforeseen effects, even if they have clinical potential. In response, the FDA issued a warning regarding the safety of unproven stem cell treatments; highlighted a number of reports of major adverse outcomes, including the development of spine tumors and blindness; and announced tighter regulation and oversight of stem cell clinics (Marks et al., 2017).

One of the primary safety concerns associated with stem cell therapy for AD is the immunological response resulting from allografting. MSCs have been demonstrated to assist in lowering immunological rejection (Di Nicola et al., 2002); however, this is still a cause for worry. Systemic immunosuppression remains the standard approach in clinical practice for lowering the chance of graft rejection, although this medication typically has a number of negative side effects in addition to possibly decreasing stem cell activity. Tobias Deuse’s team demonstrated that when major histocompatibility complex (MHC) class I and II genes are inactivated and CD47 is overexpressed, both mouse and human iPSCs lose their immunogenicity but retain their pluripotent stem cell potential and differentiation capacity, which is derived from low immunogenicity. However, genetic manipulation of stem cells to reduce or actively counteract immune rejection has also recently been performed. Treatment with mouse or human iPSCs, endothelial cells, smooth muscle cells, or cardiomyocytes consistently prevents immune rejection in fully MHC-mismatched allogeneic recipients, and patients live for extended periods of time without the need for immunosuppressive measures (Deuse et al., 2019). Secondly, threat tumor development and inadvertent differentiation. The tumorigenicity of stem cells is one of the main barriers to employing them as a foundation for regenerative medicine therapies because of their unique capacity to self-renew and specialize into multiple cell types, which is also mechanistically tied to their tumorigenic activity (Tung and Knoepfler, 2015). The case of a boy with ataxia telangiectasia who underwent neural stem cell therapy and developed a brain tumor was first reported by Gideon Rechavi and colleagues. Molecular and cytogenetic studies revealed that the tumor was not of host origin, indicating that it may have originated from transplanted neural stem cells (Amariglio et al., 2009). This anecdotal case report demonstrates that there is real danger of stem cell tumor growth and that this topic needs to be carefully evaluated. Furthermore, stem cells injected directly into the body may unintentionally differentiate into other tissues. As a result, methods such as initial differentiation into certain cells in vitro and assessment of their cellular purity to weed out undifferentiated cells should be used, as well as optimization of the stem cell differentiation technique Thirdly, additional concerns include uncontrolled in vivo environmental conditions, displacement of cells from the site of implantation, responses at the site of administration, and contaminated cells prior to injection.

### 6.2 There are no well-established SAD animal models

Sporadic AD (SAD), which affects most AD patients, is caused mostly by a combination of environmental risk factors and genetic variables. These include insulin resistance (Gupta et al., 2019; Kamat et al., 2016), inflammation (Heneka et al., 2015), hypercholesterolemia (Wu et al., 2022; Xu et al., 2020), natural aging, and brain damage (Depp et al., 2023). There is yet no animal model that can accurately depict sporadic AD disease; however, since familial AD (FAD) accounts for a tiny percentage of cases and has a well-established family history of genetic or chromosomal abnormalities, it is comparatively simple to create an animal disease model of FAD. This is among the causes of the numerous studies that have demonstrated superior outcomes in animal trials but not in human trials. Nevertheless, there are currently few animal models of SAD available for stem cell therapy (Table 3). Additionally, because most animals do not develop AD and because rodents and primates differ significantly in terms of neuronal function and anatomy, the effects of mouse models are not representative of most AD pathologies and may replicate only a limited subset of human AD pathology, not pathological features such as neuronal loss. In other words, research utilizing a variety of animal models must be performed if stem cell treatment is to effectively cure AD.

## 7 Conclusion

Since the development of stem cell technology and the capacity to differentiate stem cells into various CNS neurons and glial cells, stem cell therapy has advanced to human clinical trials and shown promise in animal models of AD. However, a full solution for AD has not been developed as yet. To properly transform stem cell research into useful applications for patients with various disorders, scientists, physicians, regulators, and ethicists must collaborate.
